# CRISPR-Cas9 disruption of PD-1 enhances activity of universal EGFRvIII CAR T cells in a preclinical model of human glioblastoma

**DOI:** 10.1186/s40425-019-0806-7

**Published:** 2019-11-14

**Authors:** Bryan D. Choi, Xiaoling Yu, Ana P. Castano, Henia Darr, Daniel B. Henderson, Amanda A. Bouffard, Rebecca C. Larson, Irene Scarfò, Stefanie R. Bailey, Genevieve M. Gerhard, Matthew J. Frigault, Mark B. Leick, Andrea Schmidts, Jason G. Sagert, William T. Curry, Bob S. Carter, Marcela V. Maus

**Affiliations:** 1 Cellular Immunotherapy Program, Cancer Center, Massachusetts General Hospital and Harvard Medical School, 149 13th Street, Room 3.216, Charlestown, Boston, Massachusetts 02129 USA; 2 Department of Neurosurgery, Massachusetts General Hospital and Harvard Medical School, Boston, Massachusetts USA; 3 CRISPR Therapeutics, Cambridge, Massachusetts USA; 4 Department of Medicine, Massachusetts General Hospital and Harvard Medical School, Boston, Massachusetts USA

**Keywords:** CRISPR-Cas systems, Receptors, chimeric antigen, EGFRvIII, Glioblastoma

## Abstract

Despite remarkable success in the treatment of hematological malignancies, CAR T-cell therapies for solid tumors have floundered, in large part due to local immune suppression and the effects of prolonged stimulation leading to T-cell dysfunction and exhaustion. One mechanism by which gliomas and other cancers can hamper CAR T cells is through surface expression of inhibitory ligands such as programmed cell death ligand 1 (PD-L1). Using the CRIPSR-Cas9 system, we created universal CAR T cells resistant to PD-1 inhibition through multiplexed gene disruption of endogenous T-cell receptor (*TRAC*), beta-2 microglobulin (*B2M*) and PD-1 (*PDCD1*). Triple gene-edited CAR T cells demonstrated enhanced activity in preclinical glioma models. Prolonged survival in mice bearing intracranial tumors was achieved after intracerebral, but not intravenous administration. CRISPR-Cas9 gene-editing not only provides a potential source of allogeneic, universal donor cells, but also enables simultaneous disruption of checkpoint signaling that otherwise impedes maximal antitumor functionality.

Glioblastoma (GBM) is the most common primary malignant brain tumor and it is also the most aggressive [1]. Despite standard-of-care multimodal therapy, over 70% of patients with GBM die within 2 years of diagnosis [2]. T-cell immune therapy represents an emerging alternative to conventional treatment, and has been shown to successfully treat solid tumors in the brain, even in the setting of bulky and invasive disease [3]. One of the most promising T-cell platforms is the chimeric antigen receptor (CAR), which has revolutionized the treatment and management of hematological malignancies with first-in-class approval by the Food and Drug Administration in 2017 [4]. However, the efficacy of CAR T cells has not been successfully translated to the setting of GBM to date [5]. One explanation for this includes the profound local and systemic immune suppression observed in patients with GBM. Moreover, autologous CAR T-cell production remains costly and time-consuming, and it can be challenging to control disease progression in GBM patients while their T cells are being manufactured. To this end, off-the-shelf CAR T cells that are resistant to local immune suppression could have meaningful benefit.

In our clinical study of intravenous CAR T cells targeting a tumor-specific mutation of the epidermal growth factor receptor (EGFRvIII) in patients with GBM, we observed that EGFRvIII CAR T cells localized to intracerebral tumors and led to successful reduction of EGFRvIII-expressing cancer cells [6]. However, this was also associated with concomitant upregulation of programmed cell death ligand 1 (PD-L1) expression within treated gliomas, ultimately contributing to immune suppression, CAR T-cell dysfunction and subsequent disease progression. In addition, four out of 17 subjects did not receive CAR T cells in the trial due to rapid disease progression, highlighting the potential benefit of “off-the-shelf,” ready-to-use products that do not otherwise require custom generation [6].

CRIPSR-Cas9 technology has emerged as a simple and efficient method of gene-editing CARs with the potential to address these barriers to therapy. This includes the design of universal CAR T cells with reduced potential for both initiating graft-versus-host disease (GVHD) and eliciting donor T-cell rejection, through targeted disruption of the endogenous T-cell receptor (*TRAC*) and beta-2 microglobulin (*B2M*), respectively [7, 8]. The use of CRISPR-Cas9 also affords the opportunity to modify the expression of other relevant genes involved in suppressing T-cell function in the microenvironment of GBM tumors.

In the current study, we applied CRISPR-Cas9 to generate an allogeneic EGFRvIII CAR T-cell product deficient in TCR and B2M. We also simultaneously disrupted endogenous PD-1 (*PDCD1*), thereby averting the potential effects of post-treatment PD-L1 upregulation in gliomas that was observed in the clinical trial. Here, we demonstrate that multiplexed gene-editing for *TRAC*, *B2M* and *PDCD1* can be performed efficiently in primary human T cells prior to CAR transduction. In addition, we observed that the antitumor efficacy of gene-edited EGFRvIII CAR T cells is enhanced by targeted disruption of PD-1 in preclinical models of GBM.

## Results

### Multiplexed gene-editing of EGFRvIII CAR T cells

**Fig. 1:**
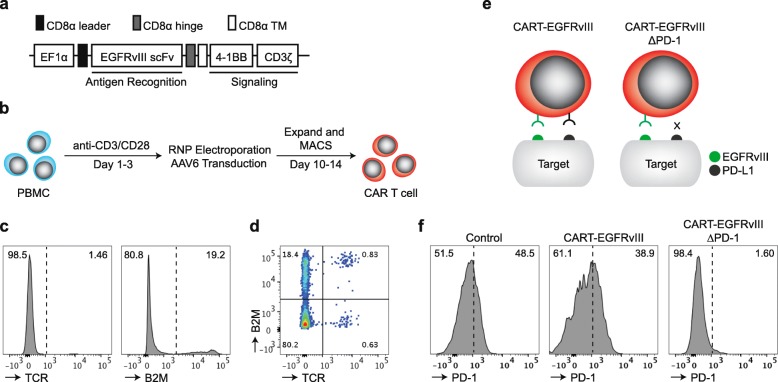
Multiplexed CRISPR-Cas9 gene-editing is efficient in primary human T cells. **a** Schematic representation of the EGFRvIII targeted CAR construct. **b** Primary human T cells were stimulated, RNP electroporated and transduced to produce CAR T cells. **c** Following expansion, cells were subjected to flow cytometry for TCR and B2M expression. **d** Bivariate plot displays frequency of cells with both TCR and B2M deletion. **e** EGFRvIII CAR T cells that have been gene-edited for PD-1 (CART-EGFRvIIIΔPD-1) do not have the ability to interact with PD-L1 expressed on target cells. **f** Effector cells were incubated with irradiated U87vIII for 1 week and subjected to flow cytometric analysis for surface PD-1 expression. The control group contains cells gene-edited for both TCR and B2M, and mock transduced with AAV

In the current study, we employed the EGFRvIII CAR T-cell construct based on a second-generation backbone containing 4-1BB and CD3ζ intracellular signaling domains, but this time cloned into an AAV6 vector backbone instead of a lentiviral vector (Fig. 1a), the former allowing for integration of the CAR sequence into a specific locus rather than relying on random genomic integration. Briefly, the strategy for multiplexed gene-editing consists of in vitro stimulation of primary human T cells, followed by electroporation with respective Cas9 ribonucleoproteins (RNPs) and subsequent adeno-associated virus (AAV)-mediated transduction of the CAR (Fig. 1b). CRISPR-Cas9 gene-editing using RNP electroporation for *TRAC* and *B2M* genetic loci was efficient, yielding populations of greater than 80% double knock-out surface expression by flow cytometry (Fig. 1c,d). In a separate experimental group, RNP electroporation was multiplexed to generate T cells also edited for *PDCD1*, in addition to *TRAC* and *B2M*. This was followed by AAV6 transduction, which resulted in CAR T cells with either endogenous or deleted PD-1 (i.e., CART-EGFRvIII and CART-EGFRvIIIΔPD-1) (Fig. 1e). Following stimulation with EGFRvIII-expressing glioma, we demonstrated that both control (i.e., T cells edited for *TRAC* and *B2M*, without CAR) and CART-EGFRvIII cells (i.e., T cells edited for *TRAC* and *B2M*, with CAR) were positive for surface PD-1 by flow cytometry. By contrast, PD-1 was not detected on the surface of CART-EGFRvIIIΔPD-1 cells, confirming effective knock-out at the level of surface protein expression in the entire population (Fig. 1f).

### CAR T-cell differentiation following CRISPR-Cas9 gene-editing

We next sought to assess the levels of PD-L1 expression on commonly used brain tumor cell lines. Importantly, PD-L1 has been shown to be frequently found on the surface of GBMs [9] and upregulated in patients treated with EGFRvIII CAR T cells [6]. To demonstrate proof-of-concept, we selected a well-characterized EGFRvIII-positive glioma line, U87vIII, as a canonical target cell for our study. Compared to its parental line, U87, and another commonly used glioma cell line, U251, we demonstrated that U87vIII naturally expresses PD-L1; however, this appeared to be decreased relative to U87 and U251 by flow cytometric analysis (Additional file 1: Figure S1).

**Fig. 2:**
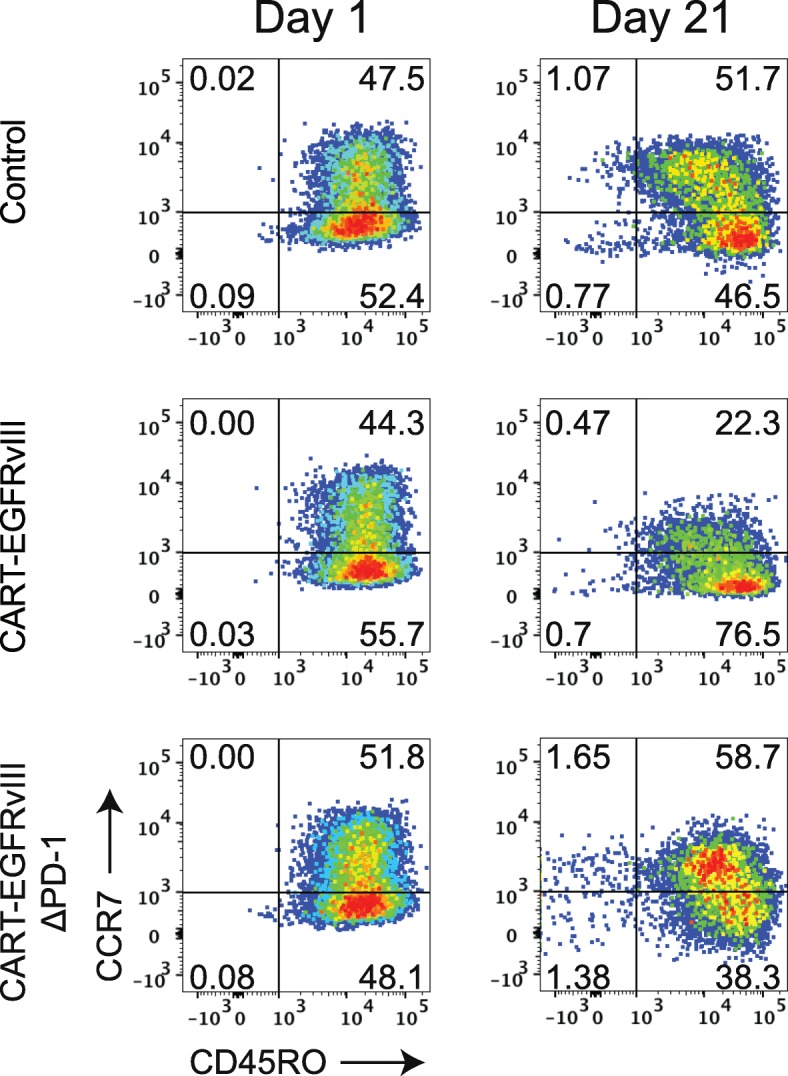
PD-1 disruption promotes favorable differentiation of CAR T cells targeting PD-L1 expressing glioma. Effector cells were cocultured with irradiated target U87vIII at and E:T of 1:1. The phenotype of T cells were assessed at Day 1 (prior to stimulation) and at Day 21 by flow cytometry. Cells were grouped by flow cytometry according to T-cell phenotype as follows: naïve (T_N_) CCR7^+^CD45RO^−^, central memory (T_CM_) CCR7^+^CD45RO^+^, effector memory (T_EM_) CCR7^−^CD45RO^+^, and effector (T_E_) CCR7^−^CD45RO^−^

We then proceeded to assess the impact of CRISPR-Cas9 gene-editing of the *PDCD1* locus in CAR T cells specific for EGFRvIII. CAR T cells are known to exist in various states of differentiation, with less differentiated stem cell memory (T_SCM_) or central memory (T_CM_) subtypes preferred over well-differentiated effector memory cells (T_EM_), specifically regarding characteristics such as expansion, persistence, and the capacity for self-renewal [10]. Moreover, loss of PD-1 has been shown to alter memory T-cell content and generation in other settings [11]. At baseline, both CART-EGFRvIII and CART-EGFRvIIIΔPD-1 demonstrated similar T-cell differentiation patterns compared to control T cells that had also been gene-edited for *TRAC* and *B2M*, in addition to undergoing mock transduction with AAV6 (Fig. 2, left column). By contrast, prolonged stimulation of CART-EGFRvIIIΔPD-1 led to a selective enrichment of T_CM_, while CART-EGFRvIII cells expressing native PD-1 appeared to enrich for the more differentiated T_EM_ compartment (Fig. 2, right column).

### PD-1 deletion promotes antitumor activity of CART-EGFRvIII in vitro

**Fig. 3:**
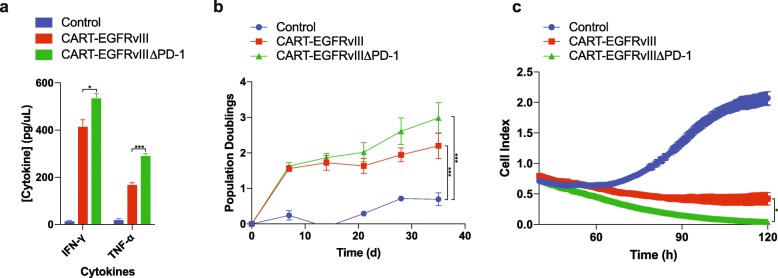
PD-1 disruption enhances EGFRvIII CAR T cells. **a** Cytokine production by CAR-transduced primary human T cells when cocultured for 18 h at an E:T of 1:1. **b** Proliferation assessment of effector cells stimulated weekly with irradiated U87vIII. **c** Impedance-based cytotoxicity assay measuring activity of effector cells against U87vIII at an E:T of 1:3, with cell index serving as an inverse measure of target cell viability. Assays were performed in triplicate (mean ± SEM is depicted; unpaired, two-tailed *t-*test, * = *P* < 0.05, *** = *p* < 0.001)

Next, we turned our attention to the functional capacity of gene-edited CAR T cells in mediating antitumor immune responses in vitro. In experiments using primary human T cells, CART-EGFRvIIIΔPD-1 cells were found to produce significantly greater amounts of Th1 proinflammatory cytokines (e.g., IFN-γ and TNF-α) when cultured with EGFRvIII-expressing glioma compared to CAR T cells expressing endogenous PD-1 (Fig. 3a). We also compared each construct for the ability to initiate and maintain T-cell proliferation. Following serial stimulation with irradiated target cells, repeated antigen stimulation through EGFRvIII maintained proliferation of both CART-EGFRvIII cells and CART-EGFRvIIIΔPD-1 cells for over 1 month (Fig. 3b). Impedance-based, microelectronic platforms were then used to capture real-time kinetics of antitumor cytotoxicity as measured by target cell index (e.g., viability). Using this system, we found that CART-EGFRvIIIΔPD-1 cells were significantly more efficacious against U87vIII than those expressing PD-1, but that this difference was observed only after an extended period of time (Fig. 3c).

### CART-EGFRvIIIΔPD-1 cells are effective against EGFRvIII-expressing glioma

**Fig. 4:**
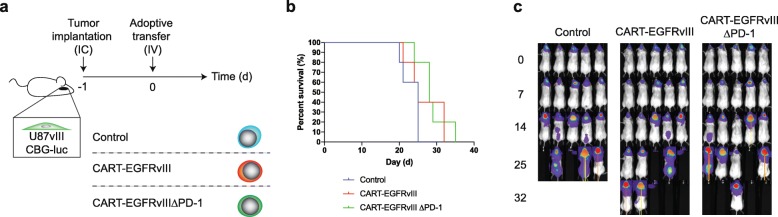
Intravenous delivery of CAR T cells does not significantly prolong survival in mice. **a** U87vIII cells (5 × 10^3^) were implanted orthotopically into NSG mice and treated post-implantation with intravenous (IV) effector cells. **b** Antitumor responses produced by CART-EGFRvIIIΔPD-1 in vivo. Survival curves were estimated for each group using Kaplan–Meier product-limit estimation. Primary comparative analyses of the curves for each group were performed using the log-rank test. **c** Bioluminescence imaging of U87vIII tumor growth over time, *n* = 5 mice

Based on our observations in vitro, we proceeded to evaluate the function of CART-EGFRvIIIΔPD-1 in animal models of human glioma. First, we implanted tumors with stereotactic assistance into the brains of NSG (NOD.Cg-Prkdc^scid^Il2rg^tm1Wjl^/SzJ) mice. This was followed by intravenous infusion of control, CART-EGFRvIII or CART-EGFRvIIIΔPD-1 cells via tail vein. Results did not demonstrate significantly prolonged survival in mice treated with EGFRvIII-specific CAR T cells systemically compared to the control (Fig. 4-c).

**Fig. 5:**
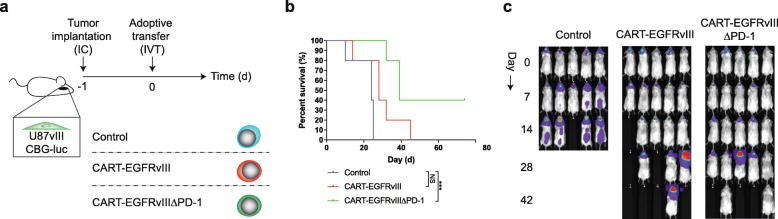
Intraventricular infusion with gene-edited CAR T cells is efficacious against GBM. **a** U87vIII cells (5 × 10^3^) were implanted orthotopically into NSG mice and treated post-implantation with intraventricular (IVT) effector cells. **b** Antitumor responses produced by CART-EGFRvIIIΔPD-1 in vivo. Survival curves were estimated for each group using Kaplan–Meier product-limit estimation. Primary comparative analyses of the curves for each group were performed using the log-rank test (*** = p < 0.001). **c** Bioluminescence imaging of U87vIII tumor growth over time, *n* = 5 mice

Because impressive results have been observed when administering CAR T cells intracranially—particularly into the ventricular system—in the setting of intracerebral tumors [3, 12, 13], we reasoned that this might also represent the ideal route for delivery of CART-EGFRvIIIΔPD-1 cells. Indeed, following intraventricular infusion (Fig. 5a), treatment with CART-EGFRvIIIΔPD-1 cells led to significantly prolonged survival in mice with EGFRvIII-expressing glioma, including durable, complete cures in select mice (Fig. 5b,c). No long-term survivors developed clinical signs of xenogeneic GVHD.

## Discussion

CARs have shown early potential in clinical trials for patients with GBM; however, treatment has been associated with marked upregulation of PD-L1 in glioma tissue, which can have profound, counterproductive effects on antitumor immunity [6]. Prior studies have demonstrated that CRISPR-Cas9 technology can be used to disrupt signaling through PD-1 in primary human T cells and to create potential “off-the-shelf,” allogeneic CAR T cell products through simultaneous editing at the *TRAC* and *B2M* loci [14–16]. In the current study, we have applied these approaches to generate universal, EGFRvIII-targeted CAR T cells resistant to PD-L1 checkpoint inhibition. In addition, we have demonstrated efficacy of these CAR T cells in murine models of human GBM. Our findings also contribute to mounting data suggesting that route-of-administration may play a critical role in achieving optimal CAR T-cell activity against tumors in the brain.

Recent work has highlighted immune checkpoint regulation through PD-1/PD-L1 as a promising therapeutic target in GBM. Aside from gene-editing techniques, a popular approach to targeting this pathway has been the use of immune checkpoint blockade (ICB) with monoclonal antibodies. Although ICB may potentially benefit certain subsets of patients with recurrent glioma [17–19], a randomized phase III study of PD-1/PD-L1 axis inhibition for GBM did not demonstrate prolonged overall survival [20]. Possible explanations for this have included concomitant chemotherapy-induced lymphopenia as well as structural considerations associated with the blood-brain barrier, which could impede interactions between systemically administered antibody and either infiltrating T cells or intracerebral tumor tissue. Unlike antibody therapies, CAR T cells have the ability to leverage profound lymphopenia to enhance antitumor activity following adoptive transfer into temozolomide-treated, lymphodepleted hosts [21, 22]. Engineering CAR T cells to secrete PD-1-blocking antibody fragments at targeted sites such as the tumor microenvironment has been proposed [23]. However, it has also been suggested that ICBs in these settings can act indiscriminately and may be responsible for hyper-progressive disease states due to unintended effects on suppressive PD-1^+^ regulatory T-cell subsets [24]. In our study, we found that deletion of PD-1 in CAR T cells, where only CAR T cells have direct cytotoxic potential (i.e., there was no antigen spreading and no secondary immune activation), had minimal effects on efficacy. Together these data suggest that GBM will require additional technologies to enhance the therapeutic effects of T-cell immunity.

Locoregional immune therapy represents a particularly attractive route-of-delivery for tumors in the central nervous system (CNS), which are thought to be isolated to some degree from the peripheral circulation by a specialized blood-brain barrier. Indeed, several studies have supported that direct infusion of CAR T cells into the ventricular system of the brain may be necessary to achieve optimal antitumor activity, and in one case this approach was required to mediate the regression of bulky, multifocal, intracranial disease [3]. Benefits of intraventricular administration into cerebrospinal fluid (CSF) spaces include enhanced access to sites throughout the CNS as well as the ability to achieve adequate effector-to-target ratios, which represents a persistent challenge of cell therapy for solid tumors [5, 25].

In this study, we applied CRISPR-Cas9 as a tool to achieve multiplexed gene-editing of human CAR T cells. Other methods of disrupting gene expression in T cells include the use of zinc finger nucleases (ZFN) [26] and TAL effector nucleases (TALEN) [27], though the use of these technologies has been relatively limited in targeting multiple genes simultaneously. Studies have shown that CRISPR can also be utilized to achieve concomitant gene integration and deletion. An example of this is a prior report of a CD19 CAR construct delivered directly into the *TRAC* locus, which also placed the transgene under control of an endogenous promoter [7]. Importantly, by virtue of these underlying mechanisms, CRISPR carries a certain risk of off-target mutagenesis. Several clinical studies are now open to evaluate the safety of this particular approach in primary human T cells; data from these trials have yet to be reported [28].

Currently, there exists a dearth of animal models that accurately recapitulate both intact immunity and antigen expression that would be encountered in a clinical setting. We elected the NSG mouse model to test our EGFRvIII CAR T cells as it permitted evaluation of a translatable human cell therapy along with the use of a human glioma cell line. One disadvantage of this approach is that it is unsuitable for experiments that seek to directly determine the efficacy of TCR and B2M deletion on GVHD or donor T-cell rejection, respectively. Ultimately, clinical trials may be the only appropriate way to definitely assess safety of these cell products in humans.

To our knowledge, this is the first report of triple deletion of *TRAC*, *B2M* and *PDCD1* in CAR T cells tested in a solid tumor model. The results obtained with CART-EGFRvIIIΔPD-1 directly address shortcomings we noted during our clinical trial of EGFRvIII-targeted CARs and thus warrant further investigation in patients with GBM.

## Methods

### Study design

In this study, we sought to apply CRISPR-Cas9 technology to EGFRvIII CAR T cells in order to address extant barriers to achieving maximal therapeutic efficacy for patients with GBM. Specifically, we created EGFRvIII-specific CAR T cells with targeted deletion of PD-1 in order to make them resistant to immune checkpoint signaling through this pathway. In addition, we used this approach to simultaneously disrupt loci corresponding to genes for both endogenous T-cell receptor (TCR) and beta-2 microglobulin (B2M). We used several preclinical modeling systems to test our hypotheses, including in vitro and in vivo platforms. These consisted of phenotypic and functional assays. Direct antitumor activity was tested against human glioma cell line targets transduced to express EGFRvIII. In this manuscript, T cells from a single healthy donor batch preparation were used, as would be used in a trial setting. Cells were not purified after genetic manipulation. The CAR T cells used in vitro were isolated from the same T-cell expansion as those used in vivo. Experiments were performed multiple times with representative data shown.

### Mice and cell lines

Immune compromised NSG mice were originally purchased from Jackson Laboratory and bred under pathogen-free conditions, according to protocols approved by the Institutional Animal Care and Use Committee. Human glioma cell lines U87 and U251 were obtained from American Type Culture Collection (ATCC) and cultured under conditions as outlined by the supplier. The U87vIII cell line was generated by lentiviral transduction.

### CAR T-cell production

CAR T cell constructs were synthesized and cloned into an AAV6 plasmid backbone. All constructs included a CD8 transmembrane domain in tandem with an intracellular 4-1BB costimulatory and CD3ζ signaling domain. Gene-editing and cell preparation was performed using standard techniques as described in detail elsewhere [29]. Briefly, human peripheral blood mononuclear cells (PBMCs) were thawed and the T cells were activated with conjugated CD3/CD28 agonists for 3 days in T-cell media containing human serum, IL-2 and IL-7. After activation, the T cells were electroporated with Cas9 protein and sgRNAs targeting the *TRAC* and *B2M* loci or *TRAC*, *B2M*, and *PDCD1* loci and subsequently transduced with a recombinant AAV6 vector containing donor template DNA for insertion of the EGFRvIII CAR construct, with a typical transduction efficiency of 35%. Following electroporation and transduction, the CAR T cells were expanded for 7 days in T-cell media containing human serum, IL-2, and IL-7. These cells were subsequently transferred to storage in liquid nitrogen prior to assays.

### T-cell assays

T-cell assays for activity, proliferation and cytotoxicity have been described in detail elsewhere [30]. Briefly, in coculture experiments, T cells were incubated with irradiated U87vIII target cells at an E:T of 1:1 for time periods as described. Cell-free supernatants from cells were also analyzed for cytokine expression using a Luminex array (Luminex Corp, FLEXMAP 3D) according to manufacturers instructions. Expression of surface markers were either taken at baseline or after a period of coculture, and then subjected to flow cytometric analysis. Antigens were stained for using the following antibody clones for flow cytometry where indicated: CCR7 (3E12, BD Bioscience); CD45RO (UCHL1, BD Biosciences), PD-1 (EH12287, Biolegend). For proliferation assays, cells were stimulated with irradiated target cells at an E:T of 1:1. Cells were counted every 7 days and plated again with stimulation at 7 day intervals. In experiments when real-time cytotoxicity was measured against U87vIII, cell index was recorded as a measure of cell impedance using the xCELLigence RTCA SP instrument (ACEA Biosciences, Inc.) according to manufacturer instructions. Percent specific lysis may be calculated from these data using the following equation: % = ((cell index of UTDs - cell index of CAR T cells) / cell index of UTDs) × 100.

### Animal models

Tumor cells were harvested in logarithmic growth phase, counted, and loaded in a 50 μL syringe with an attached 25-gauge needle. Mice were anesthetized and placed in a stereotactic frame to assist in tumor implantation. Tumor cells were implanted at 2 mm to the right of the bregma at a depth of 4 mm from the surface of the skull, in a total volume of 5 μL. Effector cells were then infused systemically by tail vein infusion in a total volume of 100 μL or administered intraventricularly in a total volume of 30 μL. Intraventricular delivery was at 2 mm to the left of and 0.3 mm anterior to the bregma at a depth of 3 mm from the surface of the skull. Effector cell populations were normalized to contain 1 × 10^6^ cells per infusion for all experiments. Tumor progression was assessed over time by bioluminescence emission using the Ami HT optical imaging system (Spectral Instruments) following intraperitoneal luciferin injection. Survival was determined by mice found expired or otherwise sacrificed by a blinded technician at predetermined humane endpoints.

### Statistical methods

All analyses were performed with GraphPad Prism 7.0c software. Data was presented as means ± SEM with statistically significant differences determined by tests as indicated in figure legends.

## Supplementary information

**Additional file 1: Figure S1.** PD-L1 expression on glioblastoma cell lines. Flow cytometric analysis of PD-L1 on human glioma cell lines versus isotype control.

## Data Availability

Data generated in this study are available from the corresponding author.

## Abbreviations

B2M: beta-2 microglobulin
CAR: chimeric antigen receptor
CNS: central nervous system
CSF: cerebrospinal fluid
GBM: glioblastoma
GVHD: graft-versus-host disease
PD-L1: programmed cell death ligand 1
TALEN: TAL effector nuclease
TCR: T-cell receptor
TRAC: T-cell receptor alpha constant
ZFN: zinc finger nuclease
